# Immobilization of *Thermomyces lanuginosus* lipase in a novel polysaccharide-based hydrogel by a two-step crosslinking method and its use in the lauroylation of α-arbutin

**DOI:** 10.1186/s40643-023-00721-9

**Published:** 2024-01-04

**Authors:** Ming Chen, Weina She, Xin Zhao, Cheng Chen, Benwei Zhu, Yun Sun, Zhong Yao

**Affiliations:** 1 College of Food Science and Light Industry, Nanjing Tech University, Nanjing, 211816 China; 2 Department of Chemical and Pharmaceutical Engineering, Southeast University Chenxian College, Jiangsu, China

**Keywords:** Arbutin, Oxidized carboxymethyl cellulose, Carboxymethyl chitosan, Microfluidic, Covalent crosslinked polymer, Lipase, Acylation

## Abstract

The *Thermomyces lanuginosus* lipase (TLLs) was successfully immobilized within a novel hydrogel matrix through a two-step crosslinking method. TLLs were initially crosslinked through the Schiff base reaction by oxidized carboxymethyl cellulose (OCMC). The water-soluble OCMC@TLLs complex was subsequently crosslinked by carboxymethyl chitosan (CMCSH) in a microfluidic apparatus to form the CMCHS/OCMC@TLLs microspheres. The CD (Circular Dichroism, CD) and FT-IR (Fourier Transform infrared spectroscopy, FT-IR) spectra demonstrated that the crosslinking of TLLs with OCMC resulted in a less significant impact on their structure compared to that with glutaraldehyde. CMCHS/OCMC@TLLs showed decreased catalytic performance due to the mass transfer resistance, while its thermal stability was greatly improved. The CMCHS/OCMC@TLLs were used to catalyze the lauroylation of arbutin in tetrahydrofuran. After 12 h of reaction under optimal conditions, the yield of 6′-O-lauryl arbutin reached an impressive 92.12%. The prepared 6′-O-lauryl arbutin has high lipophilicity and exhibits similar tyrosinase inhibitory activity and higher antioxidant activity compared to its parent compound.

**Graphical Abstract:**

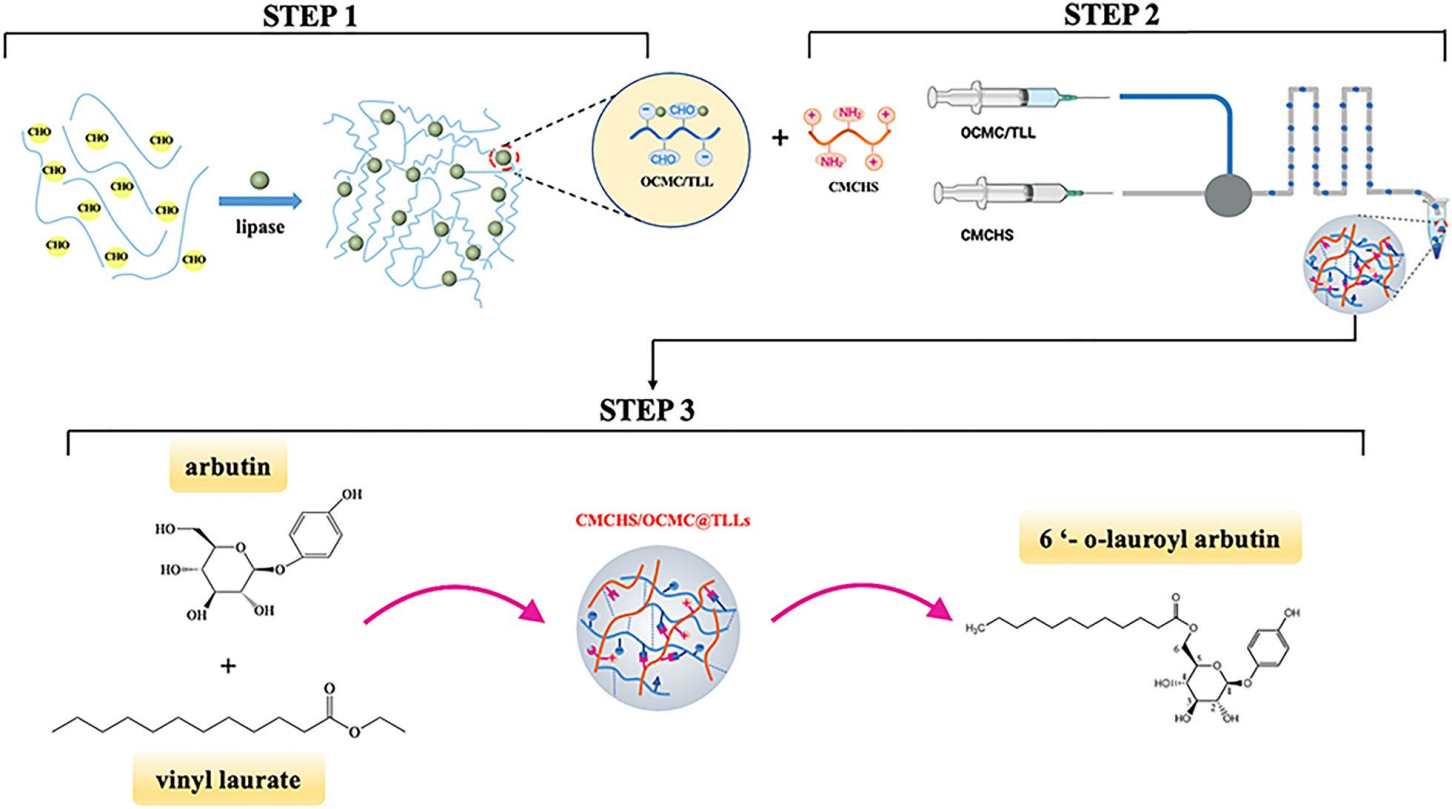

**Supplementary Information:**

The online version contains supplementary material available at 10.1186/s40643-023-00721-9.

## Introduction

Arbutin, hydroquinone O-(α/β)-D-glucopyranoside, is a natural compound found in the leaves of Ericaceae plants (Bearberry). As a member of phenolic glycosides, arbutin possesses unique biological activities of antioxidant, antimicrobial, and anti-inflammatory (Jurica et al. 2017), and anti-tumor (Takebayashi et al. 2010; Dong-Seok Kim et al. 2011; Lee and Kim 2012; Su et al. 2020; Yi et al. 2020; Nahar et al. 2022), assuming its clinical application in the treatment of urinary tract infections, kidney stones, cystitis (Schindler et al. 2002; Zhu et al. 2018), and respiratory diseases (Zhou et al. 2019). Moreover, arbutin, particularly its α-isomer has a greater inhibitory activity on tyrosinase than the β-isomer (Aung et al. 2020), serves as a potent tyrosinase inhibitor that blocks the production of melanin in vivo. Thus, it is now widely used in the production of whitening cosmetics.

From a structural perspective, arbutin consists of two components, namely, a glucoside and a phenolic group. The active Ph-OH in the phenolic group plays a crucial role in scavenging free radicals and protecting cellular constituents from oxidative harm (Watanabe et al. 2009), thus providing multiple physiological activities for arbutin. On the other hand, glucoside makes arbutin highly water soluble, but it hinders its transportation through cell membranes, resulting in a low oral bioavailability.

In an effort to solve the aforementioned issue, researchers have attempted to graft hydrophobic groups, such as long-chain fatty acyls onto arbutin by chemical (Wang and Kong 2021) and enzymatic methods (Watanabe et al. 2009; Yao et al. 2020). However, there are two active reaction sites in the arbutin structure, namely, the primary hydroxyl in glycoside and the phenolic hydroxyl in a phenolic group. It is difficult to achieve selective acylation through traditional chemical modification. Compared to chemical methods, biocatalysis offers several advantages including high regioselectivity, short reaction routes, high yields, and cost-saving benefits. Thus, the application of biosynthesis in the selective acylation of phenolic glycosides has garnered increasing attention. Tokiwa et al. (2007) synthesized arbutin undecylenic acid ester using alkaline protease from *Bacillus subtilis* as a catalyst. The acylated arbutin showed 100-fold higher inhibitory activity against tyrosinase than arbutin. Watanabe et al. (2009) studied the lipase-catalyzed acylation of phenol glycosides (i.e., arbutin, naringenin, and phlorizin) using lauric acid as an acyl donor. Lipase CALB showed a broad substrate spectrum and the yields of the resulting esters were all up to 50%. The lauroyl phenolic glycosides were more active against linoleic acid oxidation than its parent compound, probably resulting from its increased solubility and improved membrane penetration in oil-based systems. Agarwal et al. (2021) A novel amylosucrase (*AS*_*met*_) from the metagenome of a thermal aquatic habitat obtained a maximum conversion of 70% of hydroquinone to arbutin in 24 h with a sucrose acyl donor. Liu (2021) developed a novel one-step enzymatic method for the synthesis of hydrophobic arbutin esters in supercritical carbon dioxide (SC-CO_2_), using CALB as the biocatalyst and ethyl palmitate as the acyl donor (Liu 2021). The stability and solubility of the resulting esters were greatly improved, making them more useful in cosmetic and pharmaceutical applications.

Based on prior studies, immobilized lipases are preferred in the selective acylation of phenolic glycosides due to their exceptional characteristics, including a broad range of substrates, extensive source, excellent solvent tolerance, and cost-effectiveness (Milivojević et al. 2017). In recent years, there has been a significant increase in the research and development of immobilized lipase, with a primary focus on optimizing the supports (Parnianchi et al. 2018; Harris et al. 2021; Zahirinejad et al. 2021). As a host material, the supports for enzyme immobilization should possess not only high enzyme capacity but also excellent stability and mechanical strength to match different applications. In this regard, polysaccharide-based hydrogels are highly appealing due to their porous structure, non-toxicity, exceptional biocompatibility, easy modification, and biodegradability (Okura et al. 2020). The 3D mesh structure of hydrogels has the ability to both trap enzymes and allow the passage of substrates and products. Moreover, due to their highly hydrophilic nature and water-rich structure, hydrogels provide an optimal microenvironment for enzymes, thereby promoting the preservation of their conformation (Dai et al. 2017). Recently, researchers have investigated the hydrogel-based immobilization technology of lipase in detail. Park et al. (2015)immobilized *Candida rugosa* lipase in cellulose/lignin composite hydrogel beads. The immobilized lipase showed higher activity and stability than those embedded in pure cellulose beads. Kim et al. (2017) prepared bacterial cellulose (BC)–chitosan composite hydrogel beads and used to immobilize *Candida* lipase by physical adsorption and covalent crosslinking. As compared to microcrystalline cellulose (MCC)–chitosan hydrogel beads, BC–chitosan hydrogel exhibits higher adsorption capacity and activity recovery.

Despite achieving some positive results in previous work, the application of polysaccharide-based hydrogels in enzyme immobilization still faces challenges. Natural polysaccharides, for example, chitosan, cellulose, and alginate, are polyhydroxy polymers lacking highly reactive groups. During the formation of hydrogels, polysaccharide chains are commonly crosslinked through non-covalent interactions, such as hydrogen bonds and electrostatic forces. The weak interactions that exist between the chains are inadequate to ensure the structural stability of hydrogel. When exposed to solvents for an extended period, hydrogels are susceptible to swelling or even disintegration, leading to the leakage of enzymes. To stabilize the structure of hydrogel, bifunctional reagents such as glutaraldehyde and divalent metal ions Ca^2+^ are frequently used as crosslinkers to enhance the interactions between polymer chains and enzymes (Turner et al. 2005; Gu et al. 2020). However, it is possible that this small reagent scan infiltrates the enzyme structure and causes excessive intermolecular and/or intramolecular crosslinking, resulting in irreversible changes in the conformation and a reduction in enzyme activity.

**Fig. 1:**
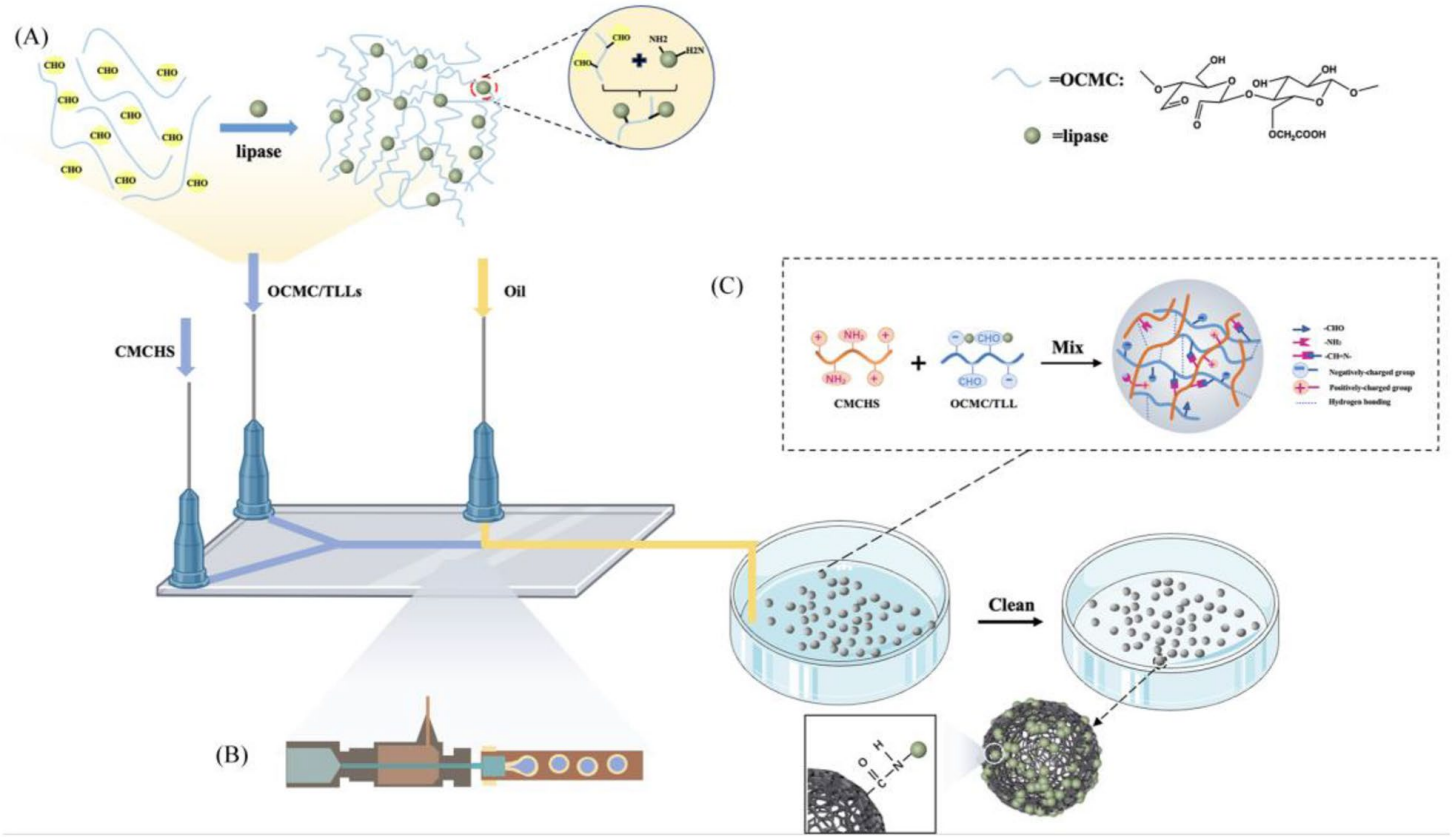
Schematic diagram for the fabrication of OCMC/CMCS@TLLs microspheres. **A** OCMC crosslinked with lipase. **B** Microfluidic fabrication of OCMC/CMCS@TLLs microsphere. **C** Crosslinking of chemical bonds within microspheres

In this study, we propose a novel method for the immobilization of lipase TLLs that involves the covalent crosslinking of oxidized carboxymethyl cellulose (OCMC) and carboxymethyl chitosan (CMCHS). First, OCMC with high aldehyde content was used to establish a chemical link through Schiff base’s reaction between the free TLLs; the OCMC@TLLs complex was subsequently crosslinked with CMCHS to yield the TLLs-containing hydrogel (OCMC/CMCHS@TLLs). To prepare hydrogel beads of uniform size, a microfluidic apparatus was utilized in this step (see Fig. 1 for the preparation route of OCMC/CMCHS@TLLs microspheres). The structure, catalytic properties, and stability of OCMC/CMCHS@TLLs were investigated in detail. In the end, we perfected a high-yield process for synthesizing 6'-O-lauroyl arbutin with OCMC/CMCHS@TLLs as a catalyst. The oil–water partition coefficient, inhibitory activity of tyrosinase, and DPPH scavenging capacity of 6'-O-lauroyl arbutin were also assessed.

## Results

### Oxidized carboxymethyl cellulose (OCMC) @TLLs complexes

#### Effect of OCMC crosslinking on enzyme activity

To improve the immobilization of TLLs, free TLLs were first linked by linear OCMC through the Schiff base reaction. The changes in activity and conformation of the OCMC@TLLs complex were investigated and compared to those of GA (glutaraldehyde)@TLLs, the complex formed by using GA as a crosslinker.

As described in 3.2.2, TLLs solution was treated with OCMC and glutaraldehyde (GA) solution, both of which had the same aldehyde concentration (approximately 0.7 mol/L), for a duration of one hour, respectively. The changes in the activity of reactants were monitored at 10-min intervals, while the activity of untreated TLLs was determined and used as a control. The initial activity of free TLLs was set at 100%.

**Fig. 2:**
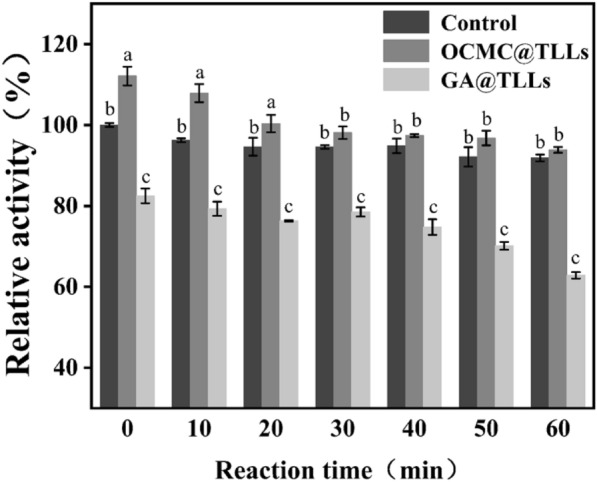
Changes in the relative activity of free TLLs, GA@TLLs and OCMC@TLLs during reaction. *a–**c**: Different superscripts (**a**–**c**) indicate significant (*p* < 0.05) difference among same group.

As illustrated in Fig. 2, the impact of GA on the activity of TLLs could be classified into two stages. In the first stage, the relative activity of TLLs decreased immediately to 82.5% in contact with GA, indicating that a rapid reaction occurred between GA and TLLs. As time progressed, the relative activity of GA@TLLs remained constant. However, a second decrease in the activity of GA@TLLs was observed when the reaction time exceeded 30 min. This phenomenon might be attributed to changes in the conformation of TLLs due to GA-induced intramolecular crosslinking.

On the contrary, the relative activity of OCMC@TLLs descended slowly during the crosslinking reaction and remained higher than that of GA@TLLs and even the free TLLs. At the end of the reaction, the relative activity of OCMC@TLLs was as high as 93.85%, whereas that of GA@TLLs was only 62.82%. This phenomenon might be explained by the special structure of OCMC. OCMC is a carboxymethylated derivative of cellulose, having a linear and flexible structure. It makes a high steric hindrance effect against the Schiff base reaction between OCMC and TLLs, which reduces the intensity of the crosslinking reaction and aids in preserving the activity of TLLs. In addition, the larger size of OCMC molecules makes it difficult to penetrate the interior of TLL. The aldehyde groups in OCMC can only react with the exposed -NH2 on the surface of TLLs molecules, thereby reducing the effect of crosslinking on enzyme conformation.

#### Effect of OCMC crosslinking on the secondary structure of TLLs

**Fig. 3:**
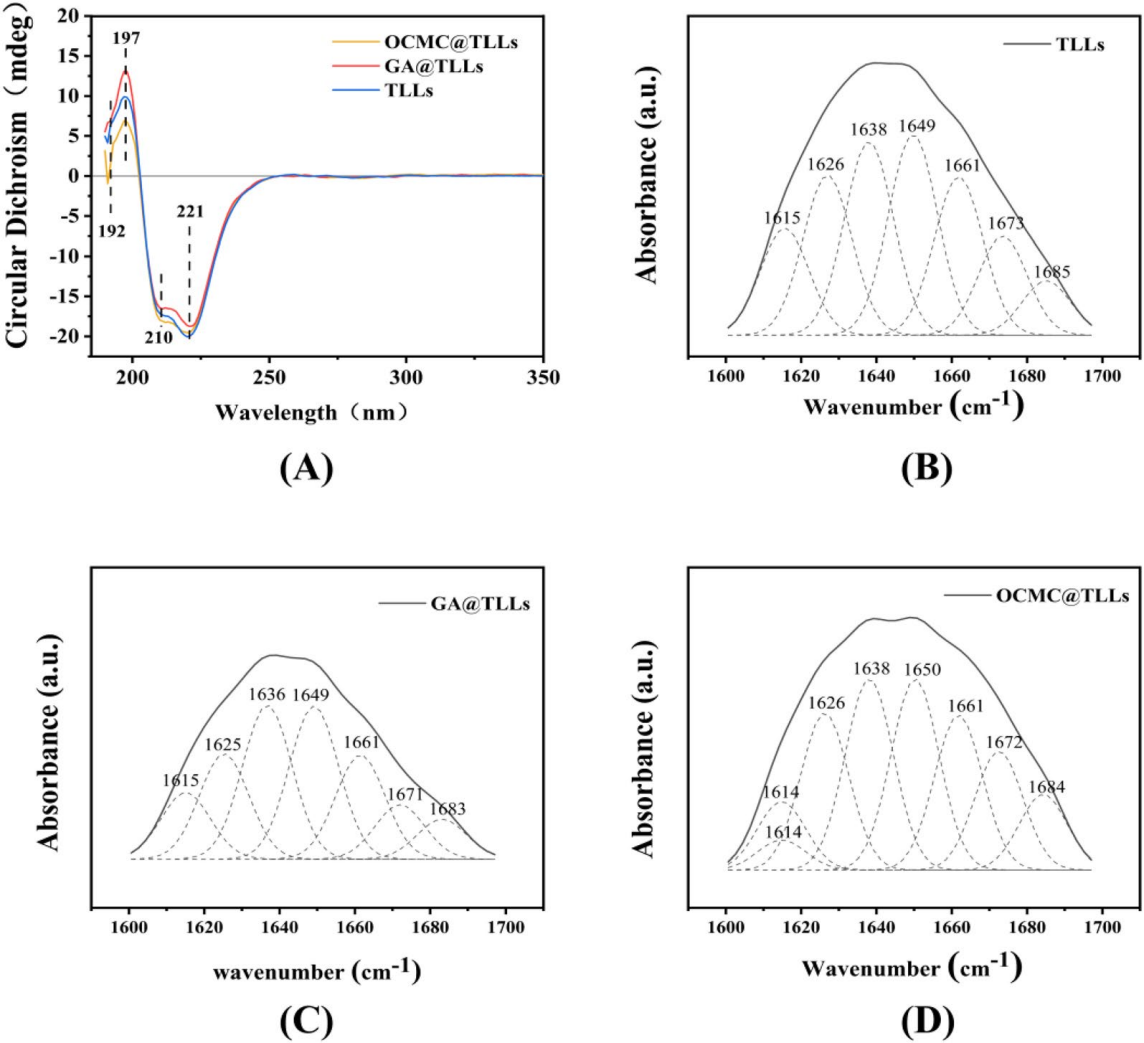
**A** Circular dichroism spectrum and deconvolution fitting of amide I region of (**B**) TLLs, (**C**) GA@TLLs, and (**D**) OCMC@TLLs

To clarify the impact of OCMC and GA on the activity of TLLs, the secondary structure of free TLLs, OCMC@TLLs, and GA@TLLs was analyzed by CD and FT-IR spectra. As shown in Fig. 3a, the CD spectrum of free TLLs presented a positive peak at 197 nm and two broad negative peaks at 210 and 221 nm in the far-UV region, which were assigned to the structures of β-sheet and α-helix, respectively (Li et al. 2021).

Compared to the free enzyme, the absorbance at 210 and 221 nm of GA@TLLs both decreased, but that at 197 nm increased simultaneously. It could be inferred that the proportion of α-helix decreased and that of β-sheet increased in the structure of GA@TLLs (Chen et al. 2021). The CD spectrum of OCMC@TLLs exhibited different changes from that of GA@TLLs, in which the positive peak at 197 nm was greatly weakened, suggesting a decreased proportion of β-sheet in the OCMC@TLLs structure.

FT-IR analysis of free TLLs, GA@TLLs, and OCMC@TLLs was done, and the curves of spectrum in the amide I band (1600–1700 cm^−1^) were fitted into their respective secondary components by means of deconvolution (Fig. 3B, D), respectively. The starting values for the center position and height of each amide I subpeak were manually fixed by inspecting each spectrum, while the width was initially fixed at 15 cm^−1^. As listed in Table 1, the proportion of α-helix and β-sheet in GA@TLLs was about 15.37% and 44.19%, which was 5.65% lower and 21.64% higher than those in free TLLs, respectively. In OCMC@TLLs, however, although the proportion of α-helix and β-sheet showed a slight decrease, the changes were not significant (< 3%). These findings were consistent with the result from the CD spectrum, confirming the distinct influences of GA and OCMC on the secondary structure of TLLs.

**Table 1:** Results of deconvolution of amide I bands corresponding to TLLs, GA@TLLs, and OCMC@TLLs

Wavenumber, cm^−1^	1624–1640 1674 –1695	1648–1660	1640–1650	1662–1686	
Samples	Secondary structure, %				
	β-sheet	α-helix	Random coil	Turn	Other
TLLs	36.33	16.29	20.66	15.76	10.96
OCMC@TLLs	35.31	15.99	19.26	19.51	9.93
GA@TLLs	44.19	15.37	22.62	8.02	9.80

### Fabrication and characterization of OCMC/CMCS@TLLs

**Fig. 4:**
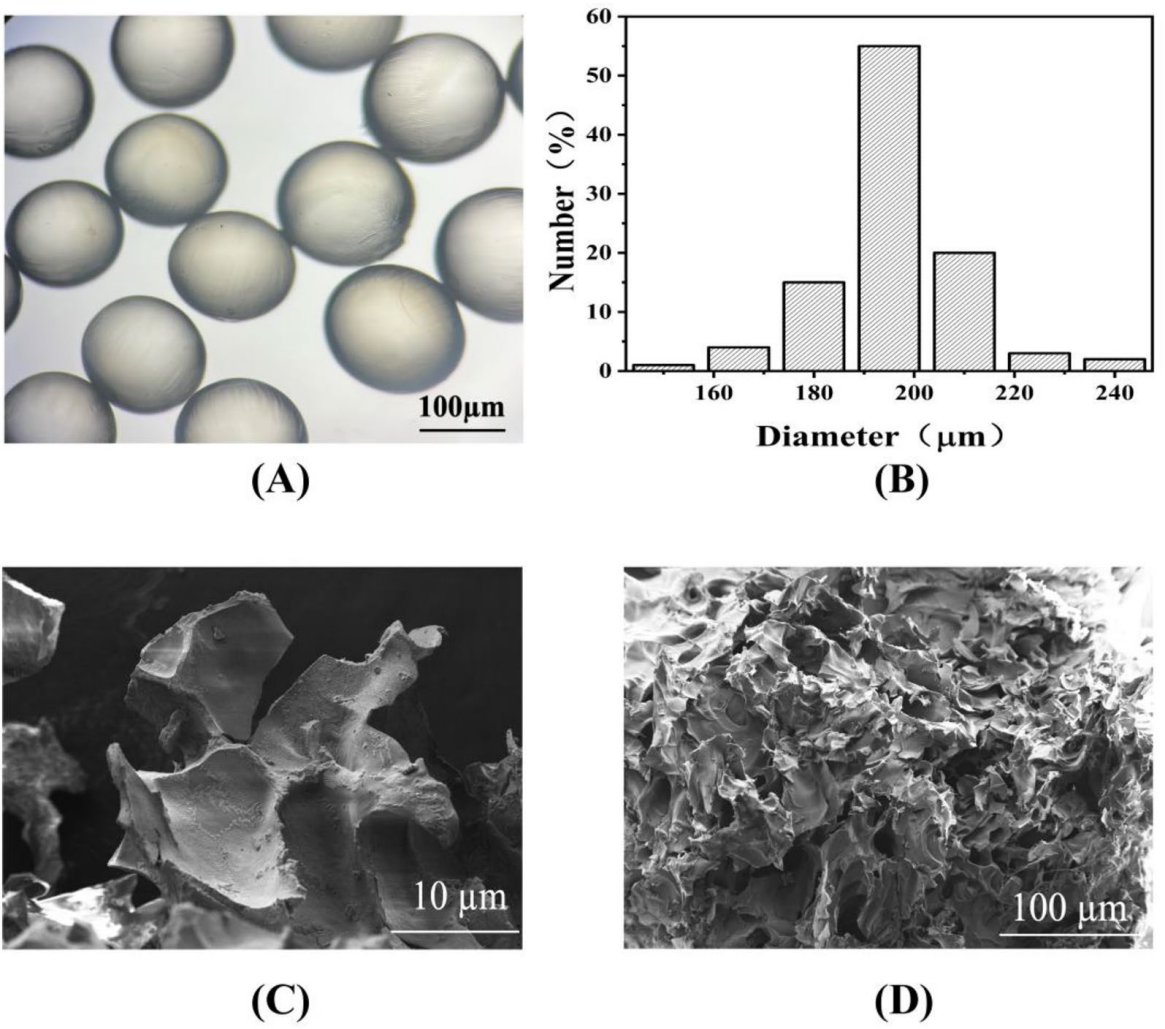
**A** The shape of hydrogel microspheres; **B** distribution of the diameter of microspheres; cross-section morphology of OCMC/CMCHS@TLLs with **C** low and **D** high resolution

The OCMC/CMCS@TLLs microspheres were manufactured using the water-in-oil method with the aid of microfluidic devices (Yang et al. 2016). The size of microspheres was regulated by altering the flow rates of disperse phase and oil phase. Upon microscopic observation, the prepared microspheres were in a spherical shape with a mean diameter of 193.28 ± 18.35 μm (Fig. 4A, B). The cross section of freeze-dried OCMC/CMCS@TLLs revealed a developed porous structure (Fig. 4D), providing a vast area for the binding of enzymes. In addition, some regular particles were observed on the surface of support from the high-resolution image (Fig. 4C), which demonstrated the successful immobilization of TLLs.

**Fig. 5:**
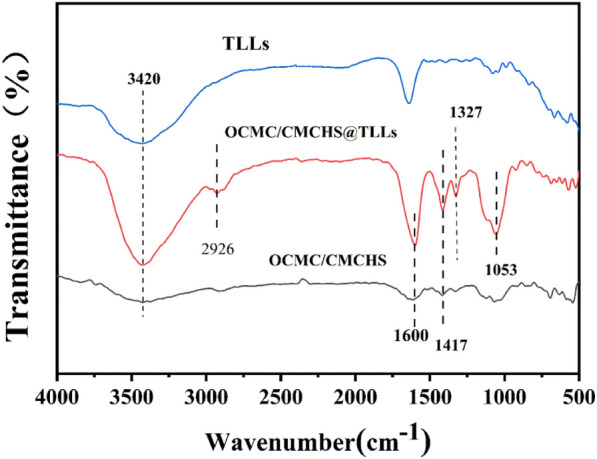
FT-IR spectra of free TLLs, OCMC/CMCHS@TLLs and OCMC/CMCHS

The FT-IR spectra of TLLs, OCMC/CMCHS, and OCMC/CMCHS@TLLs were showed in Fig. 5. The absorption peak at 3420 cm^−1^ is attributed to the stretching vibration of -NH_2_ and -OH groups. In the spectrum of OCMC/CMCHS@TLLs, the peak at 1053 cm^−1^and 2926 cm^−1^correspond to the bending vibration of C-O and the stretching vibration of C-H; 1417 cm^−1^ is the stretching vibration of -CH_2_, and the strong absorptions at 1600 cm^−1^ and 1327 cm^−1^ correspond to the characteristic peaks of C = N and C-N, respectively, confirming the generation of Schiff-based OCMC/CMCHS@TLLs.

### Enzymatic properties of OCMC/CMCHS@TLLs

#### Effect of pH and temperature

**Fig. 6:**
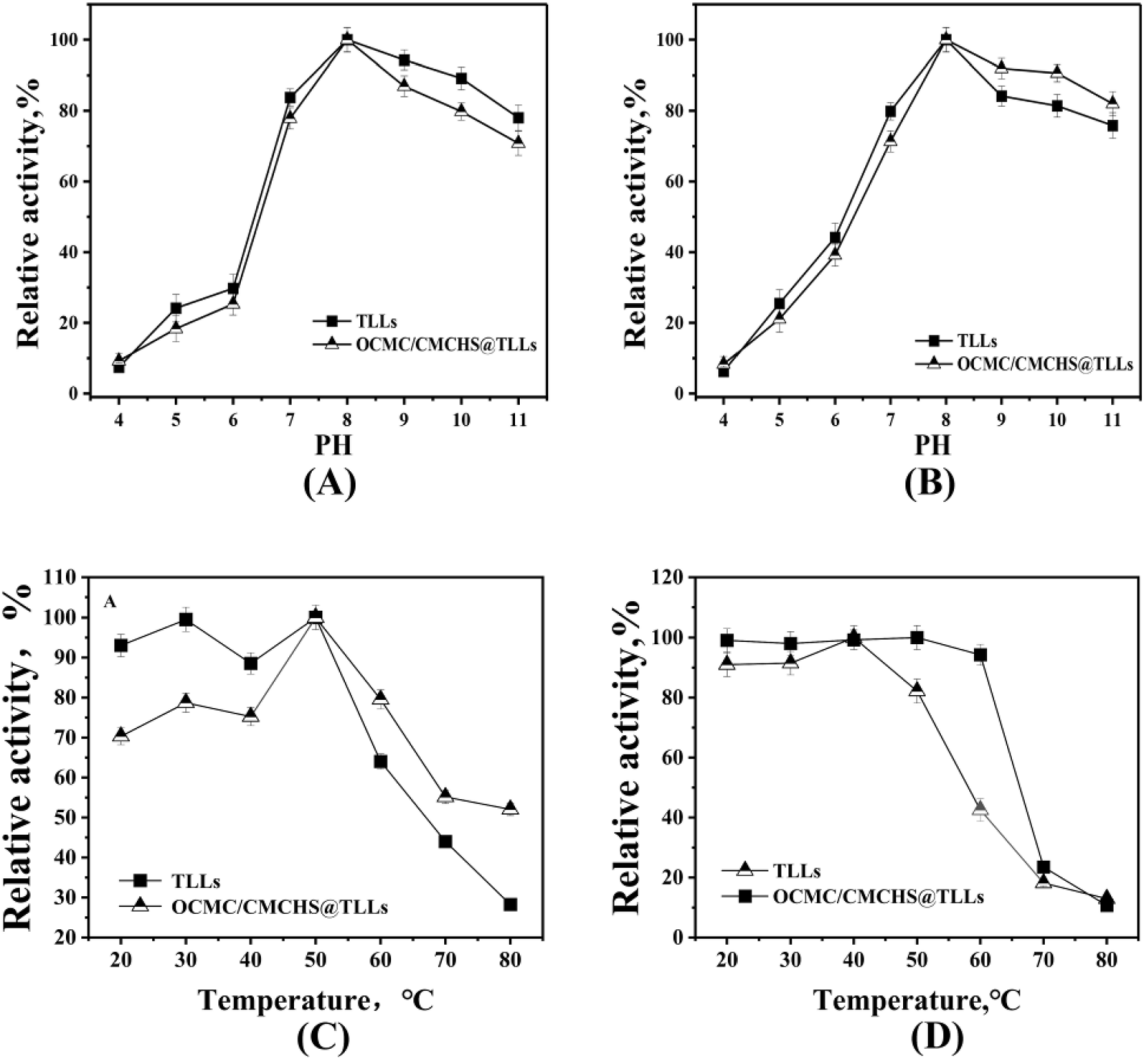
**A** The optimum pH (4.0–11.0) of TLLs and OCMC/CMCHS@TLLs **B** The pH stability (4.0–11.0) of TLLs and OCMC/CMCHS@TLLs **C** The optimal temperature (40–90 ℃) of TLLs and OCMC/CMCHS@TLLs **D** The temperature stability (60–80 ℃) of TLLs and OCMC/CMCHS@TLLs

The effects of pH on the activity and stability of OCMC/CMCHS@TLLs were examined and contrasted with those of free TLLs. In the experimental condition, the optimum pH of free TLLs and OCMC/CMCHS@TLLs was found to be approximately 8.0 (Fig. 6A). To assess the pH stability, OCMC/CMCHS@TLLs and free TLLs were separately mixed with a buffer solution of varying pH values (from 4.0 to 11.0). The samples were stood at 4℃ for 12 h, followed by assaying the activity of the samples and dividing it by its initial activity to obtain the residual activity (%). The residual activity with the highest value was established at 100%. As presented in (Fig. 6B), OCMC/CMCHS@TLLs and free TLLs were more stable in alkaline solutions (pH > 8); the highest stability for both was obtained at pH 8.0. Within the pH range of 8.0–11.0, the stability of OCMC/CMCHS@TLLs was slightly higher than that of the free form.

The impact of temperature on the activity of OCMC/CMCHS@TLLs and free TLLs is depicted in Fig. 6C. The optimal temperature for both was 50 °C. Under moderate temperature conditions (20–40 °C), the relative activity of OCMC/CMCHS@TLLs was noticeably lower than that of the free ones, which might be attributed to high mass transfer resistance in hydrogels. As the temperature increased further, the fluidity of the polymer chains in hydrogels correspondingly increased, reducing the resistance of mass transfer. In addition, the deformation of hydrogels can also mitigate the thermal denaturation of TLLs (Xu et al. 2020). Therefore, OCMC/CMCHS@TLLs displayed higher relative activity than free TLLs within a high temperature range of 50 to 80 °C.

The thermal stability of OCMC/CMCHS@TLLs was assessed by monitoring the residual activity after incubation at 60 °C for 1 h. The results presented in Fig. 6D demonstrated that the activity of TLLs remained constant within the temperature range of 20–40 °C, but it experienced a dramatic drop in stability as the temperature exceeded 40 °C. In comparison to TLLs, OCMC/CMCHS@TLLs exhibited a high level of stability across a broader temperature range of 20–60 °C. After being incubated at 60 °C for one hour, the residual activity of OCMC/CMCHS@TLLs was as high as 94.26%, confirming the protective effect of the hydrogel on TLLs activity.

#### Enzymatic kinetic parameters

The kinetics of free TLLs, OCMC@TLLs complex, and OCMC/CMCS@TLLs were evaluated by the Michaelis–Menten equation, using *p*-NPP as the substrate. By performing linear regression on $\frac{1}{{r}_{s}}$ and $\frac{1}{{C}_{s}}$, the *K*_m_ values of TLLs in three forms were calculated and are listed in Table 2.

**Table 2:** Km value of free TLLs, OCMC@TLLs, and OCMC/CMCHS@TLLs

Enzyme	Vmax mmol/ (L·min)	Km mmol/L	[E] × 10^–4^ mmol/L	kcat s^−1^
TLLs	9.597	1.396	4.21452	22,771.10
OCMC/CMCHS@TLLs	6.784	9.938	14.86367	4564.33
OCMC@TLLs	6.920	2.917	3.79307	18,243.64

The Km value of OCMC@TLLs complex was 2.917 mmol/L, whereas that of free TLLs was 1.396 mmol/L, indicating a slight alteration in the conformation of TLLs. The Km of OCMC/CMCS@TLLs was calculated to be 9.938 mmol/L, about sevenfold higher than that of free TLLs, revealing a significant decrease in its affinity for the substrate. The aforementioned results could be attributed to the high mass transfer resistance in the hydrogel. In contrast to free TLLs, the TLLs in OCMC/CMCS@TLLs were restricted within the hydrogels. During the reaction, the substrates in the solution need to be diffused through the mesh of hydrogels to the reaction site. This process resulted in high mass transfer resistance, ultimately leading to a great increase in the Km value of OCMC/CMCS@TLLs.

### Enzymatic reaction

#### Screening of the reaction media

As previously reported, the specific physicochemical properties of the reaction medium greatly influence lipase-catalyzed esterification (Garcia-Molina et al. 2010). In this study, seven organic solvents with varying solubility (Log P) and dielectric constant (ε) were tested for their suitability as the reaction medium, while OCMC/CMCS@TLLs were used as the catalyst. After a reaction at 50 °C for 12 h, the conversion rate of 6'-O-lauroyl arbutin in different reaction media was found to be in descending order, i.e., THF > tert-amyl alcohol > acetone > cyclohexane > acetonitrile > methanol (Table 3). This result could be attributed to the difference in the solubility and polarity of solvents. In the arbutin esterification system, the acyl donor and acceptor have opposite solubility. An excessively hydrophobic or hydrophilic solvent promotes the dissolution of one substrate while limiting the dissolution of the other, resulting in a decrease in the conversion rate. In addition, the polar solvents with high ε values, such as methanol and acetonitrile, may damage the structural stability of TLLs and cause the denaturation of enzymes.

**Table 3:** Conversion rate of 6'-O-lauroylarbutinin different media*

Medium	Log P	Dielectric constant, *ε*	Conversion rate, %
Methanol	− 0.76	32.6	1.11 ± 0.37
Acetonitrile	− 0.33	37.5	9.83 ± 1.91
Acetone	− 0.23	20.5	15.92 ± 0.99
THF	0.49	7.39	90.13 ± 1.55
tert-Amyl alcohol	1.3	13.35	66.31 ± 0.89
Cyclohexane	3.2	1.18	15.03 ± 1.04

*Reaction conditions: arbutin, 0.1 mmol; vinyl laurate, 0.6 mmol; solvent volume, 5 mL; temperature, 50 °C; enzyme dosage, 50 mg

#### Donor/acceptor ratio

**Fig. 7:**
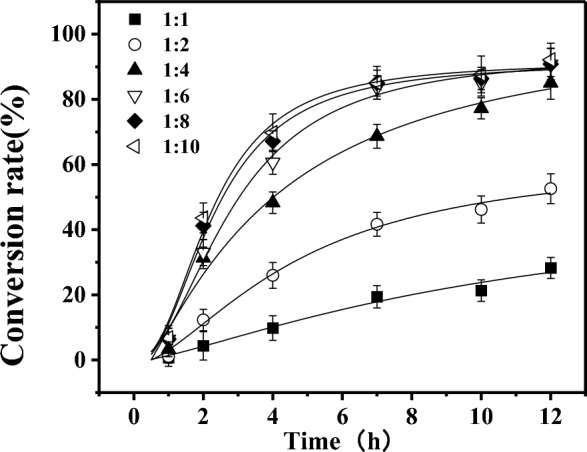
Effect of donor concentration on the conversion rate of 6'-O-lauroyl arbutin

In order to optimize the reaction conditions, a set of experiments was conducted with a fixed arbutin concentration and varying concentrations of vinyl laurate, resulting in the final molar ratios of 1:1, 1:2, 1:4, 1:6, 1:8, and 1:10; OCMC/CMCHS@TLLs was used as the catalyst with a dosage of 50 mg, and the reaction temperature was set at 50 °C. Figure 7 shows that the reaction rate and the conversion of 6'-O-lauroyl arbutin both increased with the molar ratio of arbutin and vinyl laurate. While the molar ratio was 1:6, the final conversion rate was as high as 90.79% after a 12 h reaction. Increasing the donor/acceptor ratio further had little improvement in the conversion rate and reaction time. In comparison with other relevant literature, this result was advantageous in terms of conversion rate and reaction time (Table 4).

**Table 4:** Comparison of conversion rate of arbutin with literatures

Source of lipase	Acyl donor	Medium	Reaction time, h	Conversion rate,%	Refs.
*Candida antarctica B*	Ferulic acid	tert-butanol	168	62	(Ishihara et al. 2010)
*Candida sp.* 99–1	Vinyl acetate	THF	24	91.42	(Cai et al. 2015)
*Candida parapsilosis*	Vinyl laurate	Pyridine-isooctane	24	71.1	(Li et al. 2018)
*Penicillium expanse*	Vinyl vanillic acid	tetrahydrofuran	62	93	(Yang et al. 2020)
*Candida antarctica B*	Ethyl palmitate	SC − CO_2_	20	85.21	(Liu 2021)
*Thermomyceslanuginosus*	Vinyl laurate	THF	12	90.79	This work

#### Operating stability of OCMC/CMCHS@TLLs

The operating stability of OCMC/CMCHS@TLLs was evaluated by subjecting them to a reusability test in the production of 6′-O-lauryl arbutin at optimal conditions. The same operations were conducted using TILM, commercially immobilized TLLs, instead of OCMC/CMCHS@TLLs.

**Fig. 8:**
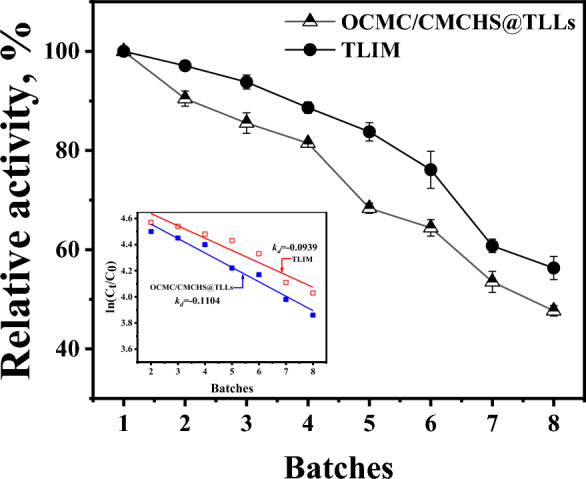
Operating stability of OCMC/CMCHS@TLLs in the reusability test

Figure 8 reveals that when using OCMC/CMCHS@TLLs or TILM as the catalyst, the conversion rate of 6′-O-lauryl arbutin gradually decreased with each reaction batch, indicating a decrease in enzyme performance. After seven repetitions, the performance of OCMC/CMCHS@TLLs and TLIM dropped to 53.52% and 60.77%, respectively. Assuming the deactivation of both OCMC/CMCHS@TLLs and TLIM in THF followed the first-order reaction kinetics. The deactivation constant (k_*d*_) can be calculated by linear regression of the following equation:

1 $${\text{ln}}\left(\frac{{C}_{i}}{{C}_{0}}\right)=-{k}_{d}\cdot t+b,$$

where *C*_0_ and *C*_*i*_ represent the conversion rate of 6′-O-lauryl arbutin in the first and batch, respectively. The calculated k_*d*_ value of OCMC/CMCHS@TLLs was 0.1104, which was slightly higher than that of TLIM, suggesting its high operating stability in THF.

### Properties of 6′-O-lauryl arbutin

#### Inhibitory activity of tyrosinase

The logarithmic value of distribution coefficient ratio, also known as the oil–water partition coefficient (Log P), is frequently utilized to determine the hydrophilicity and lipophilicity of organics. This coefficient is specific for a given compound in *n*-octanol and water. The Log P values of arbutin and 6'-O-lauroyl arbutin were determined in an octanol/water solution with a phase ratio of 1:1.

**Fig. 9:**
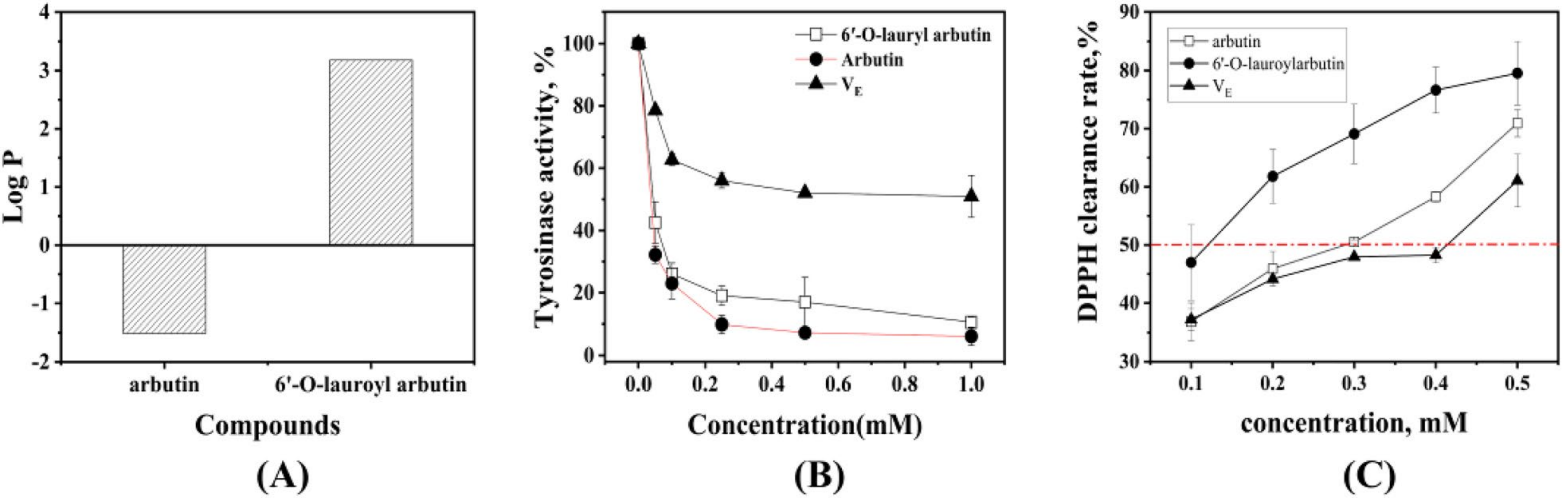
**A** The Log P values of arbutin and 6'-O-lauroylarbutin. **B** Tyrosinase inhibitory activity of 6'-O-lauroylarbutin, α-arbutin, and vitamin E. **C** DPPH radicals scavenging ability of 6′-O-lauroylarbutin, α-arbutin, and vitamin E

As illustrated in Fig. 9A, the Log P value of α-arbutin was -1.51, indicating its potent hydrophilicity; however, the Log P value of 6'-O-lauroylarbutin was as high as 3.18, demonstrating that the lipophilicity of arbutin was greatly enhanced by introducing a long-chain fatty acyl group.

#### DPPH radical scavenging

In order to examine the impacts of acylation modification on the activity of arbutin, the capabilities for inhibiting tyrosinase and scavenging DPPH radicals of both 6′-O-lauroyl arbutin and arbutin were assessed by using Vitamin E as the control. As presented in Fig. 9B, the activity of tyrosinase decreased rapidly with the concentration of inhibitors in the low dose range (< 0.2 mmol/L), but then leveled off. In the experimental range, the maximal inhibition activity of V_E_ on tyrosinase was only 50.93%, which was lower than that of α-arbutin and 6′-O-lauroylarbutin. The half-inhibitory concentration (IC_50_) of 6′-O-lauroylarbutin was 0.035 mmol/L, slightly lower than that of α-arbutin (0.041 mmol/L). This result suggested that the acylation modification on the C'-6 position of α-arbutin has a minimal impact on the tyrosinase inhibition activity.

The activity of α-arbutin and 6′-O-lauroylarbutin as anti-oxidants was assessed by the DPPH method. Figure 9C demonstrates that the scavenging rate of all three samples against DPPH radical gradually increases as their concentrations rise, indicating a distinct dose–response relationship. Compared to the control, α-arbutin showed superior radical scavenging abilities, particularly in the low dose range of < 0.35 mmol/L. Moreover, the DPPH radical scavenging ability of 6'-O-lauroyl arbutin was found to be greater than that of its parent compound. The IC_50_ value of 6'-O-lauroyl arbutin was about 0.12 mmol/L, obviously lower than that of α-arbutin. This result can be explained by the fact that the alkane chain in lauroyl is electron donating, resulting in an increase in the electron density in the oxygen of C = O. The electron-rich oxygen is likely to release electrons, thereby improving the scavenging ability of α-arbutin against DPPH radicals.

## Conclusion

In this study, we propose a novel two-step crosslinking method for the preparation of OCMC/CMCHS@TLLs hydrogel. The merits of this methodology are that, firstly, the free TLLs are initially crosslinked with a low-toxic OCMC through a Schiff base reaction, which strengthens the connection between enzymes and the carrier, thereby preventing enzyme leakage in use. Then the water-soluble OCMC@TLLs complexes are further crosslinked with CMCHS through imine bridges, created by the Schiff base reaction between the excess aldehyde groups in OCMC and the amine groups in CMCHS. This stabilizes the structural stability of hydrogels and facilitates improving the performance of OCMC/CMCHS@TLLs. The OCMC/CMCHS@TLLs microspheres were prepared with the aid of a microfluidic apparatus, which had similar enzymatic properties to free enzymes, as well as superior pH and thermal stability. Unfortunately, the catalytic performance of OCMC/CMCHS@TLLs was greatly lower than that of free enzymes due to the limitation of high mass transfer resistance in hydrogels. Moreover, OCMC/CMCHS@TLLs demonstrated remarkable performance and stability in the synthesis of 6′-O-lauroyl arbutin using THF as the reaction medium. After reacting for 12 h, the maximal yield of 6'-O-lauroyl arbutin was 92.12%, which was comparably more efficient than previous reports. The as-prepared 6′-O-lauroyl arbutin was highly lipophilic and showed nearly equivalent inhibition activity against tyrosinase as α-arbutin. In addition, the DPPH radical scavenging ability of 6'-O-lauroyl arbutin was greatly higher than its parent compound due to the electron-donating effect of lauroyl groups, which gives stronger physiological activity to α-arbutin.

## Materials and methods

### Materials

TLLs were purchased from Novozymes (Denmark). Tetrahydrofuran (THF) and CMC with a viscosity of 800–1200 mPa・s were provided by Sinopharm Chemical Reagent Co. Bovine serum albumin (BSA), Triton X-100, 4-nitrophenyl palmitate (*p*-NPP), and p-nitrophenol (*p*-NP) were sourced from Sigma. CMCHS, vinyl ester, α-arbutin, *n*-hexane, and methyl silicone oil were purchased from Aladdin. All other chemicals were acquired at analytical grade.

### Preparation of oxidized carboxymethyl cellulose

The oxidation of CMC was conducted in accordance with prior studies (Yi et al. 2020). 5.0 g of CMC and 4.28 g of sodium periodate were separately dissolved in 25 mL of anhydrous ethanol and deionized water separately. Both sample solutions were mixed up and stirred magnetically for 12 h in the absence of light. The reaction was terminated by adding 1.3 mL of ethylene glycol and continued to stir for 0.5 h. A fourfold volume of anhydrous ethanol was added to the mixture, followed by centrifugation. The precipitates were re-dissolved in deionized water and subsequently subjected to dialysis in water for 24 h. The dialyzed solution was freeze-dried to yield OCMC powder. According to Benghanem's method (Benghanem et al. 2017), the molar fraction of -CHO in OCMC was determined to be 97.2%.

### Immobilization procedure of TLLs and immobilization yield

The lipase TLLs were diluted with deionized water to a final concentration of 2.14 mg/mL, and mixed with OCMC aqueous solution (8% w/v). The mixture was stirred at 4 °C for 30 min to facilitate the formation of OCMC@TLLs complex. The aqueous solution of CMCHS (5% w/v) and the obtained OCMC@TLLs complexes were separately pumped as the dispersed phase into a Y-shaped microchip (with an inner channel diameter of 200 µm); the OCMC@TLLs-CMCHS mixture was then sheared into droplets by the continuous phase (methyl silicone oil). The flow rates of the dispersed phase were both set at 1.0 mL/min, while that of the continuous phase was 5 mL/min. The droplets flowed forward along a 0.3-m-long microchannel together with the continuous phase and solidified simultaneously. The as-prepared microspheres were collected and kept at − 30 °C for 30 min. Subsequently, they were washed with *N*-hexane and water in sequence to remove the residual methyl silicone oil and free TLLs on the surface.

The concentration of TLLs was determined using BCA protein assay kits (Biyuntian Biotechnology Company Limited, China). The p-NPP method was used to assess the activity of both free and immobilized TLLs (Akhlaghi and Najafpour-Darzi 2022) (The specific procedure is shown in Additional file 1). One unit (U) of enzyme activity was defined as the amount of enzyme required to catalyze the hydrolysis of *p*-NPP (*p*-nitrophenyl palmitate) to produce 1 μmol p-nitrophenol within one minute.

### Characterization of the biocatalysts

The morphology of microspheres was observed using an optical microscope (Nikon, ECLIPSE TS2, Japan) and a Scanning Electron Microscope (SEM, Zeiss Ultra 55, German), respectively. Fourier transform infrared spectra (FT-IR) of free TLLs, OCMC@TLLs, and CMCHS/OCMC@TLLs were obtained by a Nicolet 380 spectrometer (Thermo Fisher Scientific Co., Ltd.) at a resolution of 4 cm^−1^ in the range of 400–4000 cm^−1^. The secondary structures of free TLLs and OCMC@TLLs were analyzed by circular dichroism (BRIGHTTIME Chirascan, UK) within a scanning range of 190–400 nm.

### Enzymatic properties of OCMC/CMCHS@TLLs

#### Effect of pH and temperature

The optimum temperatures of the TLLs and CMCHS/*OCMC@TLLs *were determined at a temperature range of 20–80 ℃. To assess the enzymes' temperature stability, their retained activity was measured after incubation at temperatures ranging from 20 to 80 ℃ for 1 h. The optimal pH values for TLLs and CMCHS/OCMC@TLLs were determined under NaH_2_PO_4_–Na_2_HPO_4_ buffer solutions (pH 4.0–10.0). The pH resistance was evaluated by measuring the residual activity of TLLs and CMCHS/OCMC@TLLs after they were incubated in the mentioned pH buffers at a temperature of 4 ℃ for 12 h.

#### Enzymatic kinetic parameters

Substrate solutions (p-NPP as substrate) were configured at different concentrations (0.05–0.5 mg/mL) as described above, and lipase activity was determined as described above. The amount of product generated was calculated and fitted by the Lineweaver–Burk plotting method to obtain the constant Km of Mie and the maximum reaction rate Vmax. The lipase activity assay was repeated at least three times, and the heat-inactivated lipase preparation was used as a control instead of the active lipase preparation.

### Enzymatic reaction

The enzymatic reaction was carried out in 5.0 mL of dried tetrahydrofuran, which contained α-arbutin and vinyl laurate in varying ratios. After adding 20 mg of CMCHS/OCMC@TLLs, the reaction mixture was stirred at 50 °C and 200 rpm for 12 h, and the reaction was monitored by detecting the concentration of 6′-O-lauryl arbutin in reactant at two-hour intervals of two hours. Each experiment was performed in triplicate, and all data were calculated based on the mean values.

### Analytical methods

Quantitative analysis of α-arbutin and 6′-O-lauryl arbutin was performed by an HPLC device (Ultimate3000, the U.S.) equipped with a Venusil XBP C_18_ column (4.6 mm × 250 mm). The detection wavelength was set at 280 nm. For arbutin analysis, the mobile phase consisted of water: methanol was 80:20, while for 6'-O-lauryl arbutin, it was 20:80. The retention time of α-arbutin and 6′-O-lauryl arbutin was 4.79 min and 17.17 min, respectively.

### NMR data of arbutin mono-esters

Highly purified arbutin esters were obtained by FPLC (GE Health, the U.S.) equipped with an RP preparative column (GP-C_18_, 21.2 × 150 mm). The chemical structure of the purified monoester was determined by ^1^H and ^13^C NMR spectroscopic analysis in dimethyl sulfoxide (DMSO)-*d*6 on a Bruker 400 MHz spectrometer (Bruker, **Kurtabaugh, Germany). The detailed assignment of ^1^H NMR and ^13^C NMR signals was provided in the Additional file 1 (The spectra of ^1^H NMR and ^13^C NMR were supplied in Chapters S1, S2, and S3).

### Properties of 6′-O-lauryl arbutin

#### Inhibitory activity of tyrosinase

The inhibitory activities of α-arbutin and 6′-O-lauroylarbutin against tyrosinase were evaluated in a 96-microtiter plate (Ai et al. 2022). α-arbutin,6′-O-lauroyl arbutin, and vitamin E (positive control) were dissolved in 20%methanol aqueous solution. L-tyrosine (0.1 mg/mL) and tyrosinase (100 U/mL) solution were prepared using PBS buffer, respectively. Each well contained 80 μL of PBS buffer, 40 μL of sample solution, 40 μL of L-tyrosine solution, and 40μL of tyrosinase solution. The plate was allowed to stand at 37 °C for 30 min. During the incubation, the absorbance at 492 nm was measured at fixed intervals. The inhibition activity was calculated using the following equation (Cai et al. 2015). The blank was composed of PBS buffer without any inhibitors.

2 $$\mathrm{Inhibition\,rate }\left(\mathrm{\%}\right)=\frac{\left[\left(A-B\right)-\left(C-D\right)\right]}{\left(A-B\right)},$$

where A and B represent the absorbance value of the blank after and before incubation; C and D are the absorbance values of the sample solution after and before incubation.

#### DPPH radical scavenging

The DPPH radical scavenging ability of arbutin and 6'-O-lauroyl arbutin was evaluated using an enhanced approach inspired by Cai's study (Cai et al. 2015). The reaction system consisted of 195 μL of DPPH ethanol solution (6 × 10^–5^ mol/L) and 5 μL of the tested sample with concentrations ranging from 0 to 1.0 mM. The absorbance of the reactant was continually monitored using a microplate reader at a wavelength of 517 nm. Once the absorbance value stabilized, it was recorded. The clearance rate of DPPH radicals was calculated using the following equation:

3 $$\mathrm{Clearance\,rate }\left(\mathrm{\%}\right)=\frac{{A}_{0}-A}{{A}_{0}},$$

where *A*_0_ and *A* denote the absorbance of the blank and the sample, respectively, at the wavelength of 517 nm.

### Supplementary Information

**Additional file 1.** The spectra of ^1^H NMR, ^13^C NMR were supplied in Fig. S1, S2, and S3. The ^13^C NMR、^1^H NMR signals and structure of 6′-O-lauryl arbutin are shown in Fig. S1. Positive and negative ion mode mass spectra of purified arbutin mono lauroyl derivatives are shown in Fig. S2. The 6′-O-lauroylgeniposide structure and nuclear magnetic spectrum assignment are shown in Fig. S3.

## Data Availability

All data generated or analyzed during this study are included in this published article and its supplementary information files.
