# Supplementary material for: Immobilization of Thermomyces lanuginosus lipase in a novel polysaccharide-based hydrogel by a two-step crosslinking method and its use in the lauroylation of α-arbutin
Source: Bioresour Bioprocess. 2024 Jan 4;11(1):7. doi: 10.1186/s40643-023-00721-9 (PMC10991105; doi:10.1186/s40643-023-00721-9)
Supplement: Supplementary file 1 — Additional file 1. The spectra of 1H NMR, 13C NMR were supplied in Fig. S1, S2, and S3. The 13C NMR、1H NMR signals and structure of 6′-O-lauryl arbutin are shown in Fig. S1. Positive and negative ion mode mass spectra of purified arbutin mono lauroyl derivatives are shown in Fig. S2. The 6′-O-lauroylgeniposide structure and nuclear magnetic spectrum assignment are shown in Fig. S3. [file 40643_2023_721_MOESM1_ESM.docx]

**Additional file 1**

1.The ^13^C NMR、^1^H NMR signals and structure of 6′-O-laurylarbutin were attributed as follows:

^1^H NMR data (400 MHz, DMSO-d6):δppm9.01 (s, 1H,OH4), 6.87-6.79 (m, 2H,H2+H6), 6.68-6.60 (m, 2H,H3+H5), 5.45-5.00 (m, 3H, OH2′+OH3′+ OH4′), 4.66 (d, 1H,H1′), 4.31 (dd, 1H,H6′), 4.05 (dd, 1H,H6′), 3.60-2.98 (m, 4H,H2′+H3′+ H4′+ H5′), 2.39-2.14 (m, 2H,H2′′), 1.50 (t, 2H,H3′′), 1.23 (s, 16H,H4′′+H5′′+ H6′′+ H7′′+ H8′′+ H9′′+ H10′′+ H11′′), 1.03-0.62 (m, 3H,H12′).

^13^C NMR (101 MHz, DMSO-d6): δppm172.75 (C1′′), 152.37 (C4), 150.14 (C1), 117.64 (C2, C6), 115.42 (C3,C5), 101.52 (C1′), 76.35 (C3′), 73.64 (C5′), 73.20 (C2′), 70.03 (C4′), 63.42 (C6′), 33.53 (C2′′), 31.31 (C10′′), 29.01 (C6′′,C7′′), 28.89 (C8′′), 28.71(C5,C9′′), 28.48(C4′′), 24.42 (C3′′), 22.11 (C11′′), 13.96 (C12′′).

**
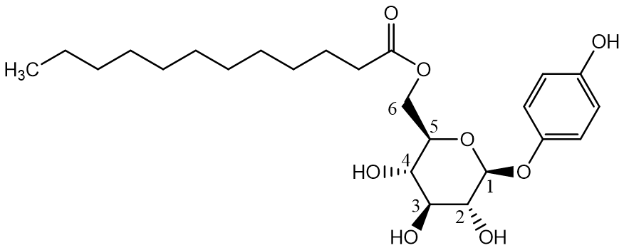
**

**Figure S1.** Structure of 6′-O-laurylarbutin

2.NMR and mass spectrometry


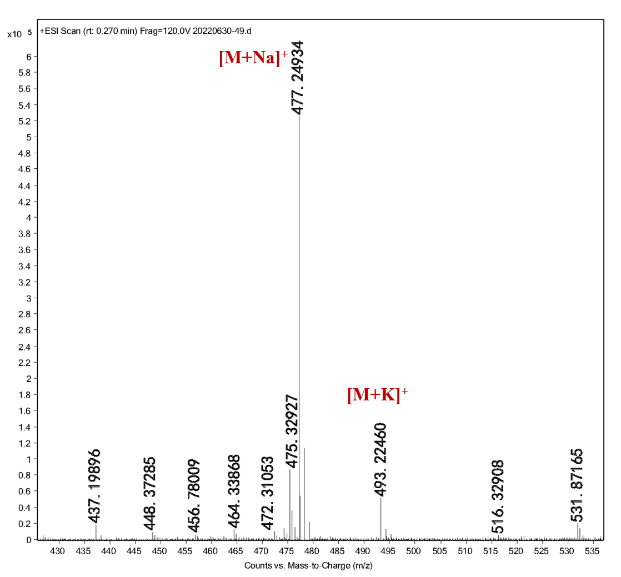

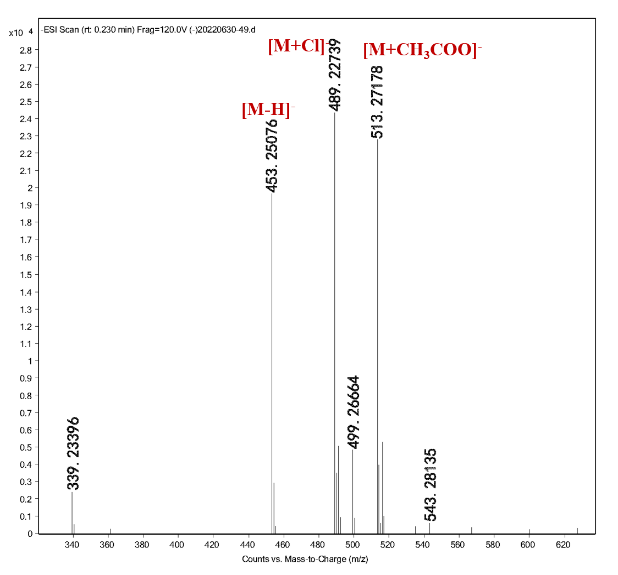


**Figure S2.** Positive and negative ion mode mass spectra of purified arbutin monolauroyl derivatives


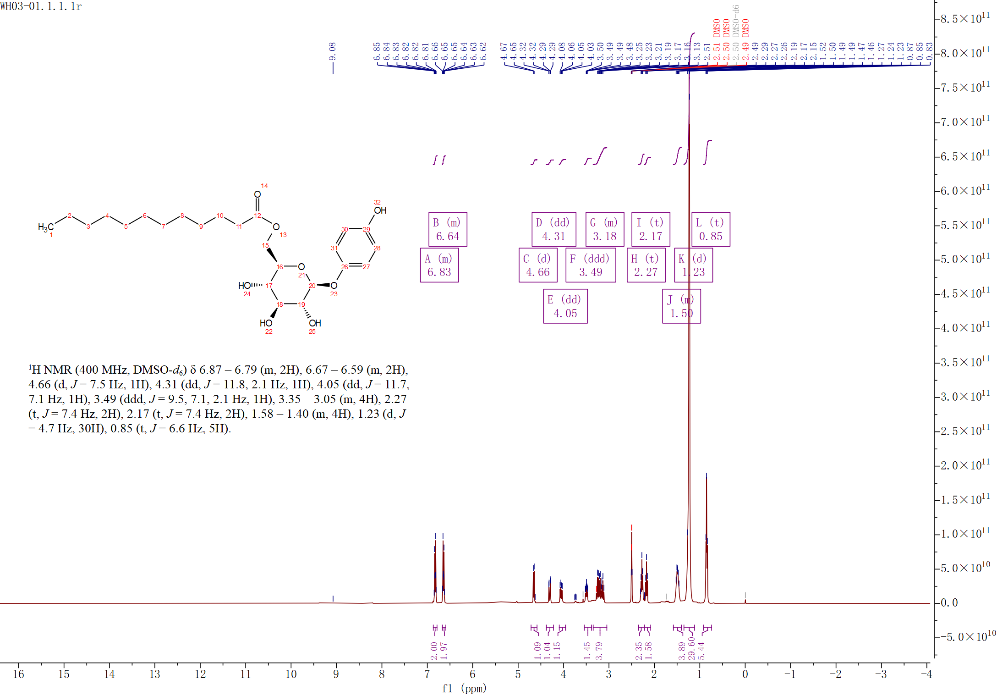


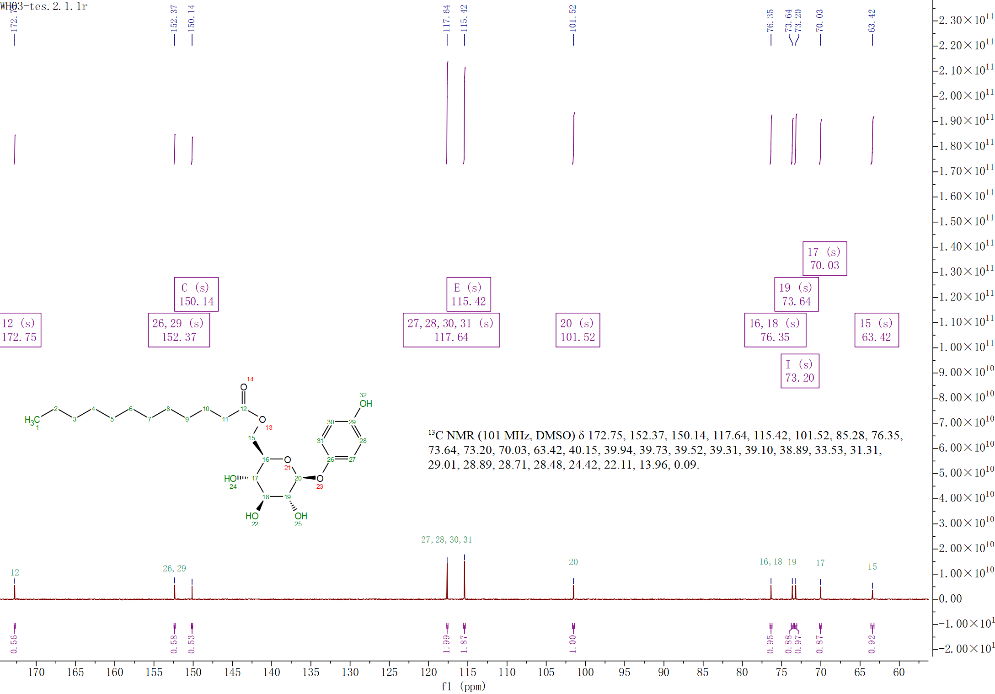


**Figure S3.** 6′- O-lauroylgeniposide structure and nuclear magnetic spectrum assignment
